# Supplementary material for: Frailty independently predicts unfavorable discharge in non-operative traumatic brain injury: A retrospective single-institution cohort study
Source: PLoS One. 2022 Oct 7;17(10):e0275677. doi: 10.1371/journal.pone.0275677 (PMC9543962; doi:10.1371/journal.pone.0275677)
Supplement: Attachment — Submitted filename: review_frail_tbi.docx [file pone.0275677.s001.docx]

The authors investigated an association of frailty in elderly patients suffered traumatic brain injury with disposition after hospital discharge. They found an association between frailty and the deterioration of outcomes. This research topic is valuable because frailty is one of the most important and urgent issues in the trauma and critical care today. However, several significant modifications are required for acceptance.

**Major comments**

1. The authors need to show the patient selection flow. Even if the study was conducted nonconsecutively, the total number of eligible patients needs to be shown first, then the number of patients who dropped out for each reason, and finally the number of patients included in the study. In addition, they should clearly indicate the number of patients excluded from the analysis due to in-hospital death.
2. I have concerns that the inclusion criteria may be inappropriate. To examine the association between frailty and unfavorable outcomes, they need to exclude patients admitted from facilities judged unfavorable. I think patients admitted from the Skilled Nursing Facility would not discharge home.
3. The authors treat the frail category as an ordinal variable. I am not sure if there is a difference between pre-frail and non-frail from this result. I perceive that the difference in the number of outcomes might just be based on differences in prehospital residence. Please clarify the characteristics of the distribution of those variables (and it would be better to exclude prehospital “unfavorable” facility). It might be better to treat frail as a binomial variable (non-frail or worse than prefrail) or as a categorical variable.
4. I recommend that variables for multivariate analysis be selected based on their clinical importance, not on their p-values. (Sun GW, Shook TL, Kay GL. Inappropriate use of bivariable analysis to screen risk factors for use in multivariable analysis. *Journal of Clinical Epidemiology*. 1996;49(8):907-916. doi:[10.1016/0895-4356(96)00025-X](https://doi.org/10.1016/0895-4356(96)00025-X))
5. In several places in the Abstract and Discussion, the authors describe primary outcomes, which are adjusted analyses, and secondary outcomes, which are unadjusted analyses, as being equivalent. I would suggest that the authors do not assert that the results of the unadjusted analysis are associated with the outcomes with the same strength as the results of the adjusted analysis.

Line 37: “Frailty was not associated with increased readmission rate, LOS, or rate of complications.”

Line 39: “Frailty is associated with increased odds of unfavorable discharge disposition but not with other major complications for geriatric patients admitted with TBI.”

Line 165: ” In a cohort of patients older the age of 70 admitted to the neurosurgical　service at a Level I trauma center, we find that physiologic frailty was associated with unfavorable　discharge disposition independently of age. We did not, however, observe a relationship between physiologic frailty and major inpatient complications, consulting services per patient, LOS, discharge GCS, or readmission/re-presentation to acute care after discharge. “

L183: “Our finding that frail patients are at risk for unfavorable discharge disposition but not other adverse outcomes”

L236: “Frailty is associated with increased odds of unfavorable discharge disposition but not with other major complications for geriatric patients admitted with TBI.”

6. Based on the journal's publication criteria, part of the conclusions would be inappropriate because it is unclear from which results they are derived.

Line 238: “Our study suggests that increased attention to the development of inpatient and outpatient care pathways and patient medical and social navigation protocols that more carefully assess alterations in neurological and physiologic derangement, and which identify and insure more complete and durable return to an optimal level of function is warranted.”

**Minor comments**

1. Line 21: The authors need to spell out “TBI”.

2. Line 22: It would be better to add elderly or geriatric patients as a target population.

3. Line 94: The authors need to define "number of consulting services provided to hospitalized patients".

4. Line 95: The citation for the definition of "major complications during hospitalization" needs to be mentioned.

5. The authors would do well to discuss the possibility that the indication for surgery causes selection bias (patients too severely ill to undergo surgery).

6. The authors need to describe that they treated GCS as categorical variables and the rationale of the category in Methods.

7. Please resubmit the Figure1 because of poor resolution.
